# Supplementary material for: Are carbon emissions trading and green financial instruments synergistic? -Comprehensive quantitative research based on content analysis
Source: PLoS One. 2024 Mar 7;19(3):e0298601. doi: 10.1371/journal.pone.0298601 (PMC10919601; doi:10.1371/journal.pone.0298601)
Supplement: S1 Data — (ZIP) [file pone.0298601.s001.zip › Data/地级市Z_F工作报告.zip!/╜¡╬≈/─╧▓2╩╨/└┤╫╘╣1⁄2╓┌║┼í╛┬φ┐╦╩2╛▌═°í┐_2005.docx]

各位代表：

现在，我代表南昌市人民政府，向大会作政府工作报告，请予审议。并请各位政协委员和其他列席人员提出意见。

一、2004年工作回顾

过去的一年，在市委的正确领导下，是我们满怀激情的一年、是我们满载辛苦的一年、是我们满市丰收的一年。我们遵循了中央的宏观调控政策，破解了制约发展的各种难题，激发了广大市民的创业热情，实现了经济发展高位增长、城市建设有模有样、三个文明协调发展、社会稳定人民安康，全面或超额完成了市十二届人大五次会议确定的工作任务。

地区生产总值770亿元，净增129亿元，增长16.5%；

财政总收入102亿元，首次超过100亿元，增长23.3%；

地方财政一般预算收入42.2亿元，增长34.3%；

全社会固定资产投资350亿元，净增115亿元，增长48.9%；

社会消费品零售总额235亿元，增长16.8%；

实际利用外资7.16亿美元，增长22.7%；

实际利用内资185.44亿元，增长37.5%；

城市居民年人均可支配收入8744元，增长12.2%；

农民年人均纯收入3414元，增长21.6%；

城镇居民登记失业率3.39%;

人口出生率13.43‰。

（一）经济发展加快，综合实力上台阶。地区生产总值净增一百亿，固定资产投资净增一百亿，财政总收入突破一百亿。主要表现在：招商引资人来客往、工业经济高位增长、农业增产农民增收、第三产业繁荣兴旺、个私经济三分天下过其一。

突出开放型经济主战略，招商引资是：重大项目多、制造业项目多、客商独资多、外商增资多。重大项目多：全市新批合同外资500万美元以上项目63个，合同外资8.38亿美元，占新批合同外资总额的80%。全市已有世界500强投资的企业11家，国内200强投资的企业22家。制造业项目多：全市新批外商投资制造业项目125个，实际利用外资3.9亿美元，占实际利用外资的55%。客商独资多：全市新批外商独资企业119家，实际利用外资4.6亿美元，占实际利用外资总额的65%。境内客商独资项目421个，占总进资项目数95%；实际进资172.96亿元，占实际进资总额的93%。外商增资多：47家外商投资企业增加注册资本，新增合同外资总额1.79亿美元，占全市新批合同外资总额的17%。

围绕打造现代制造业重要基地，工业经济是：重大政策出台、重大项目投产、重点企业壮大、园区经济增旺、产业配套增强、改制步伐加快、上市企业增加，三大指标全面实现“三年翻番”。重大政策出台：出台了“含金量”高的工业发展“三十条”，制定了制造业发展专项资金管理办法，下发了扶持光电子产业发展的若干意见。重大项目投产：奥克斯、东元电机竣工投产，晨鸣纸业即将竣工，全年共有10多个亿元以上工业项目竣工投产。重点企业壮大：全市27户重点企业完成工业增加值109.83亿元，实现销售收入381.08亿元，实现利税55.76亿元，分别占全市规模以上工业总量的61.6%、67.3%、77.1%。其中，江铃集团销售收入达到101.11亿元。园区经济增旺：11个县级以上工业园区中，共落户工业企业1111户，实现销售收入407.54亿元，增长51.3%；实现利润20.48亿元，增长14.9%；实交税金17.31亿元，增长35.3%。高新开发区实现工业销售收入124.82亿元，技工贸总收入150亿元，财政总收入5.2亿元；昌北经济技术开发区实现工业销售收入157.25亿元，财政总收入4.11亿元。接收了英雄经济开发区，完成了桑海开发区体制调整。产业配套增强：江铃整车本地化配套率达60%，家电本地化配套率达80%，饮料包装瓶本地配套率达100%，空调零部件配套建设全面推进，其中31家企业为奥克斯配套，23家企业为格林柯尔配套。改制步伐加快：加强国有资产管理，成立了市国资委；市直国有（集体）工业企业147户中，已有70户企业完成改制。上市企业增加：洪城水业在上交所、行知软件在香港创业板成功上市，分别募集社会资金2.75亿元和3000万港元。江西纸业成功复牌，多年债务顺利化解。目前，南昌地区已有上市公司16家。三大指标实现“三年翻番”：全市规模以上工业企业实现工业增加值、销售收入、利税，均在2001年基础上实现三年翻番。

坚决落实党的“多予、少取、放活”政策，农村经济是：粮食产量超历史、经济结构更优化、基层基础在夯实、农民收入创新高。粮食生产：粮食总播面491.8万亩，增长20.7%；粮食总产34.8亿斤，增长20.8%；平均亩产768斤，增长15.5%。经济结构：推进农业综合开发，形成一县一业开发格局，拥有无公害农产品基地36个，面积57.5万亩。成功举办了首届南昌生米藠头节、第二届军山湖螃蟹节，军山湖大闸蟹远销海外；安义丛玉蔬菜被中央警卫局定为人民大会堂蔬菜特供基地。粮食作物优质品率达90%，生猪良种率达90%，水产品优质品率达76%，特种水产品占水产品总量达32.7%。市级农业产业化龙头企业50家，其中，省级龙头企业17家、国家级龙头企业6家、年产值超亿元的龙头企业12家，17家企业41个品牌获得国家级绿色产品认证。基层基础：实施农村扶贫项目和农村“双带”致富工程，落实扶贫资金1144万元，“双带”致富项目信贷资金2877万元，技术对接项目108个，“双带”致富村支书已达720人；农村公路水泥路全年完成1000公里，1177个行政村有1155个已通公路；完成重点公益林保护面积41.4万亩、营造林面积22.54万亩；完成37座圩堤双退、2座圩堤单退任务，68座病险水库除险加固正在实施，修建“小山塘”、“小门塘”2000座；鄱阳湖二期治理工程、赣抚大堤加固工程等重点堤防建设进展顺利；解决1.6万农村人口饮水困难。农民收入：粮食直补、良种补贴和农业税调减3个百分点的政策全部落实到户，全市发放粮食直补和良种补贴共计8762万元；取消农业特产税使农民减负859万元，调减农业税使农民减负7096万元，实现了农民负担零税赋（农业税和补贴持平）。全市农民年人均纯收入达3414元，增长21.6%，超过城镇居民收入增幅；农民人均纯收入中，粮食收入929元，比上年多收入363.5元，增长64.2%。

着力“做旺人流、激活物流、融通资金流”，第三产业是：商贸流通繁荣活跃、房地产业稳步提升、现代服务不断拓展。在商贸流通上：特色街喜添新贵、大卖场又增新宠、大物流再创新高。特色街：在现有8条商业特色街的基础上，新建了江大南路商业街。大卖场：世界500强麦德龙所属青山湖商场隆重开业；百货大楼销售额18.3亿元，增长20.5%；洪城大厦销售额13.2亿元，增长37.5%。大物流：交易额超亿元商品市场发展到30个，洪城大市场年交易额118.2亿元，增长9.2%；南昌（深圳）农产品批发市场年交易额28.4亿元，增长8.8%。整顿规范市场经济秩序，查处假冒伪劣商品案值650万元。在房地产业上：土地经营张弛有度、房屋开发稳健推进、低洼危房改造加快、房产交易市场活跃。全市招拍挂出让土地1378亩，新增融资16.38亿元。新建房屋完成施工面积1068万米2，经济适用房30.7万米2；商品房交易面积388.93万米2，交易额95.55亿元，增长17.6%和21%；存量房交易面积215.4万米2，交易额29.35亿元，增长39.8%和38.3%。在旅游会展上：旅游产业份额提高、质量提升，会展经济端倪初现、亮点频显。全年游客达到592.58万人次，旅游总收入39.2亿元，增长14.9%。成功申报“梅岭——滕王阁”国家重点风景名胜区，天香园“都市候鸟”载入上海基尼斯纪录。中国南昌第三届金秋经贸活动月期间，英雄城南昌节日一个连一个、客人一批又一批、商家一队接一队，天天是节日的气氛，处处是欢乐的景象。共举办大型活动19场，参与人数350万人次，来昌客商近万人，签约项目171个，合同外资1078亿美元，合同内资6826亿元。在现代服务业上：金融保险持续发展，信息服务增势强劲。截止12月底，全市金融机构各项存款余额为1316亿元，增加185亿元；各项贷款余额为873亿元，增加101亿元；各类保险机构保费收入18.56亿元，增长18.4%。上海浦东发展银行开业准备工作进展顺利。中国南昌政府网站走在全国前列；全市家庭宽带网覆盖率为7.5%，城市百人拥有移动电话87部，农村百人拥有移动电话50部。

坚持“放开、平等、服务”，个私经济是活力四射，潜力强劲：个私经济增加值达260.96亿元，占全市GDP比重由上年的31.3%提高到33.9%；增加值净增62亿元，占全市GDP增量的48%。

（二）建设步伐加快，花园城市初具规模。朝着“蓝天常现、碧水常流、绿色常驻、春色满城”目标，坚持“五高”，注重“四增”，四脉传承，四网并举，完善设施，强化功能。

一是整治天上的线网、打通地面的路网、完善地下的管网、沟通城市的水网。线网：强电弱电，统一规划、合理布局、逐片规范，上杆、下地、进管，做到整齐美观；昌西、艾溪湖、双港、麦园变电站投入运行，电网承载能力明显增强；路网：提升出城大路，沟通城中道路，整治小街巷路，启动环城高速，新建生米大桥。出城大路：按照“10分钟到主干道、20分钟到外环线”的要求，改造迎宾大道，新建红湾公路，扩建（昌进、南安、省大、南高）四条一级公路，完成国道、省道改造77公里，基本建成市至县半小时经济圈公路网络。城中道路：经路纬路、大路小路，改造延伸、连通成网。小街巷路：完成了第二轮百路大会战，改造道路112条。环城高速：启动了东、西外环绕城高速公路。生米大桥：完成工程设计量的50%。管网：对供水管、下水管、排污管、燃气管，实施同沟共井工程，新修道路，一次布管、同时下地；道路改造，一次开挖、多管齐下。水网：增强供水，处理污水，治理积水，活化水系。增强供水：青云水厂三期工程如期竣工、牛行水厂一期工程开工建设，新建昌东、罗家增压泵站，新建改造城市输配水干管100余公里，初步形成南至莲塘、东至罗家、西至湾里、北至乐化的城市供水体系；处理污水：完成占地300亩、投资3个亿、一期日处理污水33万吨的青山湖污水处理厂，启动红谷滩新区4个污水泵站项目建设，筹建象湖污水处理厂、红谷滩污水处理厂；治理积水：增大排水管网、沟通排水体系、增加排灌机组、实行雨污分流，解决城市内涝；活化水系：截污流、清淤泥，畅通东西两河（桃花河、玉带河），沟通外四湖（象湖、青山湖、艾溪湖、梅湖）。

二是调整城区区划、理顺管理体制、构建物流平台、强化公共服务。城区区划：按照划清界限、便于管理、有利发展的原则，以路、河、湖为界，调整界定了东湖、西湖、青云谱、青山湖四个城区区划，解决了老城区多年来界线不清、职责不明、纠纷不断、扯皮不停等问题。管理体制：完善了“二级政府、三级管理、四级网络”管理体制，成立了市城市管理行政执法局，在西湖区开展了街居管理体制改革试点。物流平台：开通南昌——深圳、南昌——厦门海铁联运，与上海、宁波共建无水港，新建国际集装箱码头，启动铁路西站，南昌兴发物流开始试营业；公共服务：完善功能、提升水平。新增公交线路9条，延伸8条，调整11条，新增公交线路营运里程213.45公里；新增高速客运班线8条，延长营运里程3050公里；徐坊一级长途汽车站竣工投运。

三是挖掘文化底蕴、装点江河湖泊、提升城市品位。八一广场：以八一精神为灵魂、以纪念塔为标志，经历三个寒暑春秋，奋战六百日日夜夜，八一广场顺利竣工。改造后的八一广场融继承性和开创性、纪念性和休闲性、广场主题和周边环境于一体，更加宽广壮丽、更加庄严雄伟、更加特色鲜明。城市绿化：新栽乔木42.2万株，灌木1260万株，铺草213.3万米2，新增绿地284公顷，城市绿化覆盖率达36.88%，人均绿地7.1米2。象湖：截断污流换活水，叠起重山植万树；打造楼台敬贤圣，翰墨诗赋入画图；龙舟竞渡小舟渡，万人垂钓万人舞；水中荷花岸边柳，一城春色半城湖。梅湖：调转五干水（渠），开通城南河；碧波荡梅湖，青云谱青波。玉带河：主支、西支、北支，原来是：洪都排渍道，纵横四十里；脏乱加破烂，污水伴污泥；人道龙须沟，半点不差厘。现在是：拆迁数千户，万人大转移；桥涵几十座，管线过百里；跨道穿“京九”，雨污两分离。将来是：借来洪门水，豫章生灵气；乞取西山树，绿色染大地；鱼翔蛙声伴，蝶飞百鸟啼；英雄城画卷，人民铸丰碑。

（三）文明程度提高，各项事业协调发展。围绕创建“四城”，实施文明行动，加强民主法制，协调各项事业，促进可持续发展。

实施文明南昌行动：全面打响“五大文明工程、创建平安南昌、完善社会保障、市容环境整治”四大战役。五大文明工程：文明行风，要求做到：待人不冷、不燥，做事不推、不拖、不卡、不索；文明表率，要求做到：举止端庄、表率风范、严于律己、善谋实干；文明市民，要求做到：不乱吐、不乱扔、不乱窜、不乱爬；文明社区，要求做到：安全防范、邻里和睦、整洁卫生、健康快活；文明村镇，突出抓好“六个一”：一根线（农村用电）；一条路（乡村公路硬化）；一口塘（农村用水）；一座山（植树绿化）；一个村（村容、村貌、村风、村规）；一批校（改造危房，确保安全）。措施是：统一指挥、相互协调、落实责任、倾听热线、每周调度、及时整改、加强督导、责任追究、排名排位、新闻曝光。现在的南昌，正朝着人要文明礼貌、车要各行其道、整治楼道和路道而不懈努力。创建平安南昌：突出三项安全，开展专项整治，制定应急预案。全市没有发生大规模群体性事件、严重暴力性案件、重大治安事件、恶性火灾事故、重特大安全生产事故，维护了社会稳定、确保了一方安宁。完善社会保障：严格落实“两个确保”，重点帮助下岗职工、城镇低保户、农村五保户。城镇新增从业人员7.62万人，下岗失业人员再就业3.3万人。提高低保标准，实现应保尽保。市区由165元/人·月提高到190元/人·月，农村县区由125元/人·月提高到150元/人·月；全市享受低保人数10.59万人，共发放低保金8244万元，增加2083万元。农村五保户做到了“摸清户头、落到人头、雪中送炭、疏而不漏”。全市共发放社会救助资金3116万元，增加713万元。在青云谱区进行了失地农民养老保险试点。市容环境整治：重点是抓好三大项（市民行为、交通秩序、楼道路道），整治三专项（建筑工地、集贸市场、六小场所），攻克三难点（垃圾、污水、噪声），突破一重点（除四害），管住四门（进城口、城乡结合部），抓好三带一资料（灯光带、绿化带、碧波带、创卫资料）。一年来，清理卫生死角1.3万处，拆除违章建筑22万米2，修补道路5.26万米2，取缔占道摊点6.92万个（次），“六小整治”卫生合格率60%以上，“五车”整治帮扶对象1070人，整治农贸市场82个，油烟净化率达92.2%，排污达标率90%以上。

加强民主法制建设。落实行政许可法，取消行政审批121项，保留359项，为全国最少的城市之一；认真贯彻执行《南昌市优化投资环境条例》，进一步打响“四最”品牌；完善了一站式服务、办证绿色通道和审批办证首席代表制、行政委托制、告知承诺制；认真受理外商投诉，投诉解决率达100%。2004年台湾权威商业机构评定：南昌为中国内陆地区十大最具投资吸引力城市之一。提请市人大常委会审议地方性法规草案5件，制定规章5件，清理规章43件，报请省政府备案的规范性文件13件，办结行政复议案件61件。认真执行人大及其常委会的决议，自觉接受人大法律监督、工作监督和政协民主监督。坚持领导干部星期日学法制度，加强普法教育，开展法制援助；加大惩治腐败、纠风治乱、政府效能建设力度，勤政廉政水平得到提高。政府各部门、各执法单位能够文明、公正执行公务，取得明显成效。

协调发展各项事业。教育：继续加强义务教育，实施优质高中工程，推进农村学校危房改造。小学、初中入学率为99.4%和97.1%，高中毛入学率为70%，高考录取率为79.65%。南昌科技大学新校区一期工程全面竣工，入驻学生6000余人；南昌二中红谷滩新校区、中国人大附中南昌实验学校、京东安居小区九年制学校开工建设；龙泉小学基本完工；接管省属企业学校13所，接管铁路学校11所；南昌已成为全国三大职业教育基地，打造了“学在南昌”品牌。科技：加大科技成果转化力度；成功加入世界科技城市同盟（WTA），成为全国第10个加入该组织的城市；“国家半导体照明工程产业化基地”顺利落户南昌，成为全国四大基地之一；全省首家高新技术投资风险公司——“南昌创投”挂牌运作。人才：落实人才绿卡制度，完善人才吸纳措施，加强人才培养力度。已建两家博士后工作站。选派了105名党政干部赴新加坡、澳大利亚学习培训。卫生：万人拥有病床34.94张，万人拥有医护人员48.97人。大力实施“三名工程”，首批认定名科10个、名医12人。加强疾病控制，强化卫生监督，健全社区卫生网络，推进农村医疗试点；疾病控制中心、紧急救援中心动工建设；引进民营医院9家，市三医院改制全面启动。文化、广电：加强对外文化交流，南昌采茶戏应邀赴澳门演出，喜获成功。 精心打造三条楹联文化街，成功举办首届楹联文化节，来自海内外的400多名联界精英会聚南昌，泼墨写天下、落笔走烟云，规模之大、范围之广、水平之高，勘称楹联史之最；南昌被授予全国首座楹联文化省会城市。经几代人共同努力，文港镇喜获“华夏笔都”荣誉称号。满载着丰收的喜悦，伴随着激扬的鼓点，成功举办“长征组歌”和“中华情·激情南昌”大型文艺晚会。全市第三次文物普查工作顺利完成。体育： 奥运得金牌，农运省第一，承办老运会，举办市运会，万人健步行（第28届奥运获得金牌1枚；在第五届全国农运会上，获得金牌4枚、银牌2枚，名列全省第一）。

促进可持续发展：人口与计划生育：持续稳定低生育水平，提高出生人口素质，加强计划生育文化宣传，提高计划生育优质服务水平。资源保护：严格土地审批程序，规范土地市场管理，落实耕地保护政策，确保耕地占补平衡，通过了清理检查验收。加强水资源管理，开展“保护母亲河”行动。环境保护：强化环境污染防治，严格项目环境审批、实施创建国家环保模范城“六大工程（蓝天、碧水、宁静、绿化、固废、宣教）”，改善生态环境质量。饮用水源水质达标率为99.1%，空气优良率为90.1%。

同时，加强国防动员、民兵预备役和人民防空工作，0112工程全面竣工，加强军民共建，荣获全国双拥模范城“五连冠”。加强统战、对外联络、外事侨务、对台、接待、民族、宗教、参事、地名、档案、史志、老龄、工会、共青团、妇女、儿童和残疾人等工作。

各位代表：过去的一年，南昌是：起早摸黑辛苦耕耘的一年，三个文明喜获丰收的一年，科学发展夯实根基的一年。

一年来，我们的工作体会是：

——突出开放，做强“底子”。坚持大开放，开展大招商，主动对接长珠闽，积极融入全球化，知名企业落户南昌，外资、内资流入南昌，做强了南昌的“底子”。

——突出经济，做大“盘子”。坚持让工业、农业、商贸三产、个私民营齐头并进，让劳动、知识、技术、管理、资本竞相迸发，壮大经济总量，提升经济质量，做大了南昌的“盘子”。

——突出环境，做美“样子”。朝着“五高”目标，注重“四增”举措，拉开城市框架，强化城市功能，打造一流人居环境，提升英雄城市品位，做美了南昌的“样子”。

——突出文化，做响“牌子”。弘扬传统文化、创新现代文化、丰富群众文化，加强文化交流、发展文化产业，让世界了解南昌，让南昌走向世界，做响了南昌的“牌子”。

——突出文明，做足“面子”。坚持以人为本，关注人民生活，实施文明行动，全力创建四城，推进民主决策，严格依法行政，做足了南昌的“面子”。

各位代表，上述成绩的取得应归功于党中央、国务院方针政策的指引，归功于省委、省政府的正确领导，归功于省直部门的大力支持，归功于社会各界的热情帮助，归功于全市上下的共同努力、拼搏进取。在此，我代表南昌市人民政府，向为南昌经济发展和社会进步作出贡献的全市工人、农民、干部和知识分子，致以崇高的敬意！向各民主党派、工商联、群众团体、社会各界朋友和驻昌解放军指战员、武警官兵、公安干警，表示衷心的感谢！

二、2005年目标任务

2005年，是贯彻落实党的十六届四中全会精神、全面建设小康社会的关键之年，是加强宏观调控、全方位推动改革开放的重要之年，是落实省委、省政府“一年有个新变化、三年有个阶段性变化”要求的交帐之年。现在的南昌正面临千载难逢的机遇：经济全球化，给南昌带来了参与国际分工的广阔商机；城乡一体化，给南昌带来了加快产业融合的发展平台；承东启西、沟通南北的区域优势，给南昌带来了全方位对接“长珠闽”的巨大空间；“人心思上、人气兴旺、经济提速、城市变样”，给南昌带来了率先崛起的坚强基础。但是我们也必须清醒地看到面临的困难和问题：经济总量还不大，支柱企业还不多，配套能力还不够，城市管理水平还不高，城市服务功能还不强，社会就业压力还较大，城市居民收入还不高等等。对此，我们要高度重视，认真解决。我们坚信，南昌人民誓将闻鸡起舞，腾蛟起凤，奋书崛起风流；扬帆起航，劈波斩浪，尽显英雄本色。

今年工作的总体要求是：

以“三个代表”重要思想为指针，全面贯彻党的十六大和十六届四中全会精神，按照省委、省政府加快南昌发展的要求和市委八届八次全会确定的工作部署，坚持科学的发展观和正确的政绩观，紧紧围绕发展第一要务，全力做好两篇文章，切实做到六个坚持，确保实现六个提高，即：坚持扩大开放、深化改革，提高发展活力；坚持统筹兼顾、三产并举，提高经济实力；坚持规划先行、建管并重，提高城市品位；坚持以人为本、关注民生，提高生活水平；坚持和谐协调、全面发展，提高文明程度；坚持公平公正、服务至上，提高行政效能。

今年工作的主要预期目标是：

地区生产总值900亿元，增长14.5%；

财政总收入117.3亿元，增长15%；

地方财政一般预算收入47.7亿元，增长13%；

全社会固定资产投资440亿元，增长25.7%；

实际利用外资8亿美元，增长11.8%；

实际利用内资208亿元，增长12.2%；

社会消费品零售总额270亿元，增长14%；

城市居民年人均可支配收入9650元，增长10%；

农民年人均纯收入3756元，增长10%；

城镇居民登记失业率控制在4.5%以内；

人口出生率控制在13.54‰以内。

（一）坚持扩大开放、深化改革，提高发展活力。

坚持开放主战略，做好改革大文章，主动对接长珠闽，积极融入全球化，消除发展障碍，激活发展细胞，释放发展能量。

开放：坚持城门大开，十里相迎，诚招天下客，善待来昌人。争取国家资金，用好信贷资金，吸引外来资金，激活民间资金，融通社会资金，筹措企业资金；扩大消费领域，优化消费结构，减少消费壁垒；完善出口激励机制，落实出口退税政策，用好“海铁联运”平台，营造出口通关优势。让投资、消费、出口“三轮”驱动，让劳动、知识、技术、管理、资本活力迸发。招商引资重点做到“五个联动”：优惠政策和优越环境联动：落实各项优惠政策、完善外商办证绿色通道、切实帮助解决实际问题、加强治安专项整治，营造“四最”品牌优势。政府主导和市场主体联动：今年政府将实施新一轮“百、千、万”招商计划（百场招商活动、千批次小分队、接洽万名客商），全面推进工业、农业、三产服务业和社会事业等领域的招商；大力发展以商招商、以外引外、以企引企，充分调动企业、中介机构招商引资积极性，促进政府招商向市场主体招商转变。产业招商和区域招商联动：发挥南昌区位优势，紧盯产业梯度转移，对接长珠闽、突出港澳台、拓展日韩欧美，盯住莱索托，开展针对性招商。招大引大和增资扩股联动：瞄准重点企业、重点人物，力争引进世界500强企业1—2个，国内200强企业2—3个，同时支持入驻企业做大做强，争取增资扩股。项目落户和项目见效联动：加强项目跟踪服务，促进项目早签约、早进资、早开工、早投产、早见效。

改革：借助外力，启动内力，凝聚合力。全面推进国企改革，大力发展非公有制经济，加快企业上市步伐；加强国有资产监管，推进事业单位改革。国有企业：积极引进战略投资者，形成投资主体多元化、混合型所有制、法人治理结构合理、企业资产和职工身份实现“双置换”的国有企业经营发展新格局。非公有制企业：完善中小企业服务体系，设立中小企业发展资金，扩大非公有制经济份额。企业上市：力争全年新增上市企业1—2家，募集资金2—3亿元。国资监管：健全国有资产监督管理体系，完善市国有资产监管机构，落实国有资产经营责任，促进国有资产保值增值。事业单位：按照行政管理职能与资产管理职能、资产管理职能与资产经营职能、公益性资产和经营性资产分开原则，推进市政公用设施、社会事业领域改革。县域经济：按照“能放权就放权、能少管就少管、能不管就不管”原则，扩大县区经济管理权限；调整完善县、乡财政管理体制，加大财政支持力度，增强县乡财政实力，增加县域经济发展后劲。农垦企业：加快农垦企业转机建制步伐，加大对农垦企业扶持力度，使农垦企业重焕活力。

（二）坚持统筹兼顾、三产并举，提高经济实力。

以打造现代制造业重要基地为核心，调整优化现代工业；以基地化、特色化、产业化为重点，稳定提升农村经济；以增强城市综合服务功能为目标，创新提升第三产业；做大经济总量，做优经济结构，做强经济实力，加快建设现代区域经济中心城市步伐。

现代工业：突出园区主战场，提升企业竞争力，形成产业集聚群。一是在园区经济上：完善功能配套、调整产业布局、强化土地集约。功能配套：搞好基础设施，完善市政配套，畅通信息物流，健全服务体系，打造多功能、高效率、低成本的商务平台。产业布局：南昌高新开发区重点发展电子与信息、光机电一体化、新材料、生物医药产业；南昌经济开发区重点发展现代家电、汽车及机械制造、电子信息、现代造纸产业；桑海开发区重点发展医药产业、保健品；其他各类园区要立足现有优势，找准产业定位，壮大支柱产业。土地集约：盘活土地存量、争取用地增量，提高存量土地利用率、提高单位土地面积投入产出效益。二是在优强企业上：争取政策、扶持龙头。争取国家项目立项，争取省里技改贴息；重点扶持：江铃、江中、南烟、南钢、洪钢、洪都航空、泰豪科技、济民可信、奥克斯、东元电机等40家重点企业；力争全年技改投入70亿元。三是在产业集聚上：强化政策引导、加强专业协作、推进产业配套，重点发展汽车、空调、光电和软件、食品、造纸、飞机制造、纺织服装、医药和医疗器械、机电产品、建材和新材料十大产品制造基地。

农村经济：围绕提高农业综合生产能力，加强农村基础设施，加大支农扶农力度、调整优化经济结构、稳步推进三大转移，确保增产增效增收。一是加强农村水、电、路等基础设施建设：完成各大圩堤和74座水库除险加固，大力兴建山塘水库；加强农村电网建设改造步伐，保障农村发展需要；加快退耕还林步伐，加强森林防火设施建设；加快县道、乡道、村道建设，确保村村通公路、力争村村通客运。二是加大支农扶农力度：稳定、完善和强化扶持农民、农业、农村的各项政策，确保政策不变、力度不弱、实惠不减。从今年起，全市一律免征农业税。同时，加大转移支付力度，法定增长支农资金，继续实施种粮、良种、农机“三补贴”，进一步完善农村基础设施、农业技术推广、农产品市场开拓三大支撑体系。三是调整优化经济结构：按照“山上办绿色银行，山下建优质粮仓，水面兴特色养殖”思路，建设优质稻生产基地，培育壮大翘嘴鳜、珍珠、花卉苗木、无公害蔬菜、食用菌、小水果、河蟹、藠头、奶牛、藜蒿十大基地，推进基地化；按照种养区域化、生产标准化、品种专业化要求，打造“一乡一业、一村一品”特色，推进特色化；按照“一头连市场，一头连基地、公司加农户”模式，推进产业化。四是稳步推进三大转移：按照“自愿、依法、有偿”原则，稳步推进土地向大户转移，提高农业生产效率；按照“城镇带动、城乡一体”方向，稳步推进人口向城镇转移，提高农民非农化水平；按照“集约化经营，产业化发展”思路，稳步推进企业向园区转移，提高农副产品加工企业的市场竞争力。

第三产业：提升传统商贸，做大物流平台，改善流通环境，拓展服务领域。一是提升传统商贸。运用先进技术，发展新型业态，改造提升商贸、餐饮等传统服务业，做大百货大楼、洪城大厦、新洪客隆等龙头企业，打响特色街品牌。二是做大物流平台。大力发展物流配送，规划建设昌南、昌北、昌东、昌西现代物流园。三是改善流通环境。农村大力实施“绿色通道”工程，畅通农副产品流通渠道；城市全力实施“食品药品放心”工程，大力开展专项打假，切实维护消费者权益。四是拓展服务领域。稳定提升房产地产，繁荣活跃旅游会展，规范发展中介组织，大力发展金融保险。房产地产：规范引导市场、盘活土地存量，增加经济适用房指标，落实住房货币化政策；房产交易总额120亿元。旅游会展：按照“海内海外连成网，景点宾馆一条龙”模式，整合文化旅游资源，打响“红色八一、古色千年、绿色生态、沙漠绿洲、都市候鸟”旅游品牌，力争2005年旅游业收入50亿元，增长27.5%；按照“规范管理，培育市场，打造品牌”思路，做大会展经济。中介组织：放宽市场准入、加强秩序管理，有序发展会计、律师、咨询等新兴服务业。金融保险：引进金融旗舰，做大保险龙头，培育资本市场，打造信用南昌。

（三）坚持规划先行、建管并重，提高城市品位。按照“加速昌北、提升昌南、启动朝阳”建设格局，朝着“五高”，注重“四增”，传承“四脉”，完善“四网”，彰显“一江两岸、南北两城、滨江依湖、文化名城”特色。昌北要：东沿赣江摆开，西揽梅岭入怀；孕育山水灵气，传承豫章文化；体现现代气派，汲取世界精华；构筑大板块、打造大昌北、建设大都市。南昌是一支笔写出人杰地灵，一杆枪打得天下扬名。昌南要：挖掘文化底蕴，打响英雄品牌，保护都市候鸟，改善人居环境，再现“滕阁金塔、双峰并峙”之雄浑、“东湖月夜、南浦飞云”之妩媚。朝阳要：加大基础投入，强化功能配套，构架路网，沟通水网，建设电网，完善气网。（昌北两河五湖七库：乌沙河、玉龙河，前湖、黄家湖、碟子湖、礼步湖、白水湖，溪霞、乐化、幸福、乌井、梦山、肖峰、朱坊水库；昌南两河八湖：抚河、玉带河，东西南北湖、象湖、青山湖、艾溪湖、梅湖）。今年城市建设政府融资65亿元，重点抓好十大工程：一是城市框架工程：抓紧外环、改造中环、打开出城通道、建设环城高速。城市外环：完成昌东大道二期工程；城市中环：完成高新南大道二期、青山湖大道一期、青山路大道工程，凤凰北大道、红谷北大道、红谷南大道、蝶子湖中大道、阳明路东延、赣江南大道延伸段工程；筹划赣江穿江隧道。出城道路：完成机场景观路，开工昌进二期、昌万一级公路、莲塔超二级公路56公里，完成国省道公路改造61公里，消灭干线砂石路，全面建成半小时经济圈公路网络；环城高速：西外环高速公路全线路基拉通，完成生米大桥建设。二是城市防洪工程：完成赣江两岸70公里防洪圩堤的除险加固，管好现有六座电排站，兴建鱼尾闸电排站，规划、筹建邓家山电排站。三是城市交通工程：沟通东西、畅通南北、完善路网、增加设施、划清标线，改善城区交通拥堵现象。四是港口建设工程：完成南昌主枢纽港总体规划编制及上报审批，上半年完成南昌国际集装箱码头建设。五是供水净水工程：完成牛行水厂建设，新建、改造长堎水厂、昌北、湾里地区供水主管网，加快象湖、红谷滩污水处理厂建设。六是供气供电工程：加快新建城区、新变电站及输配线路建设，加快老城区居民低压电网建设，完成桃苑、京东、象北、福山、船山、万溪、广场变电站建设，确保用电高峰期居民用电和工业用电需要。完善城南、城东现有管道煤气供气，加快朝阳地区、红谷滩新区管道液化气建设。七是城市管网工程：增设地下管网，实现雨污分流；规范空中线网，电线、电话线、电视线、电缆线，精心清理、逐片规范。八是市容环境工程：加强硬件，强化软件，开展市容环境集中整治行动，重点整治集贸市场、在建工地、小街小巷、六小店面、四门七路城乡结合部，做到：街街整治，店店美容，户户袋装，人人保洁；街面做到：绿化、亮化、美化，不脏、不乱、不暗，晚上好走，白天好看。完成红谷滩新区ISO14001环境管理体系认证工作。确保创建国家卫生城顺利过关。九是河湖治理工程：装点水面，美化沿岸，打造豫章明珠，呵护城中瑰宝。整治玉带河中支、东支，提升西支、北支、主支，启动前湖景区建设，加快两河八湖水系水质污染自动监测系统建设。十是美化亮化工程：新增绿地面积100公顷，人均公共绿地达7.2米2，路灯增至9.5万盏，路灯完好率100%。建设梅湖公园、朝阳公园，加快动物园搬迁。总之，使南昌“绿色常驻、春色满城”。

（四）坚持以人为本、关注民生，提高人民生活水平。注重基层，看到底层，同情可怜人，帮助困难人，关心残疾人。坚持“贴近市民、关爱百姓、带着感情、饱含深情”，做到“一枝一叶总关情”。重点是：扩大就业、强化保障、提高收入、民心工程。扩大就业：通过发展经济开发岗位、发放贷款创造岗位、政府补贴提供岗位、培训技能争取岗位，提高就业比率。力争实现新增就业岗位6万个，安置下岗失业人员1.5万人，转移农村劳力16万人。强化保障：扩大养老保险、失业保险、医疗保险、工伤保险、生育保险覆盖面，确保离退休人员养老金百分之百按时足额发放，确保国有企业下岗职工基本生活保障向失业保障并轨，平稳过渡到位。确保城镇低保户应保尽保，农村五保户、特别困难户生活有保障。加大农民工资清欠力度，切实维护农民务工权益。同时，完善价格预警机制，合理稳定物价水平，确保人民生活安定。提高收入：着力提高低保特困户综合生活质量，适当提高财政拨款工作人员收入水平。民心工程：围绕增加就业、改善医疗、环境整治、农村扶贫、安全饮食、改善交通、社会服务、改善居住八个方面，安排专项资金，用于补助项目建设，全力推进今年的20件民心工程项目建设。

（五）坚持和谐协调、全面发展，提高文明程度。遵循全省“建设和谐平安江西，共建富民兴赣大业”主题教育活动要求，结合南昌实际，全力打造南昌有序竞争的创业环境、公平公正的执法环境、和谐安宁的社会环境。重点是：深化文明创建，发展社会事业，维护社会稳定，促进可持续发展。文明建设：继续实施文明创建工程，加强文明教育，强化规范管理，达到“人人规范自己行为、个个争当文明市民”。社会事业：重点是创新科技体制、构筑人才高地、优先发展教育、繁荣文化广电、发展卫生事业、提高体育水平。科技：突出区域创新体系建设，建立并用好市级科技发展专项资金和科技成果转化专项资金，加快多元化、多渠道科技投融资体系建设，推进产学研结合，加速科技成果转化；人才：抓住人才引进、培养、使用三个环节，建设高素质公务员队伍、高素质专业技术人才队伍、高素质企业经营管理人员和高素质技能人才队伍，再建两家博士后工作站。教育：优化义务教育网点布局，做大做强高中阶段教育，整合提升职业教育资源，全力打响“学在南昌”教育品牌。推进农村中小学危房改造工程、优质高中建设工程、城区九年制义务教育示范学校工程，加快新二中、城东地区九年制义务教育示范学校建设；启动市委党校新校区建设。文化、广电：挖掘文化底蕴、发展文化产业、抓好文化载体、丰富文化生活、打响文化品牌。启动八一起义纪念馆扩建工程，加快推进铁军广场、八大山人书画陈列馆、市博物馆、市民俗博物馆新馆等文化设施建设。卫生：健全公共卫生体系、加大卫生执法力度、提高卫生保健水平，继续抓好农村新型合作医疗试点工作。加快市疾病预防控制中心、南昌急救救援中心、市第九医院感染病综合大楼建设。体育：普及群众性体育活动，提高竞技体育水平。启动新体育中心、丹霞体育公园和市体校整体改造工程。社会稳定：重点是确保四项安全：坚持“打防结合、预防为主、专群结合、依靠群众”的方针，加强社会治安综合治理，妥善处理各类矛盾纠纷，构建和谐平安南昌。坚持“安全第一、预防为主”，加强安全生产，落实专项治理，确保生产、交通、消防、食品安全。增强国防观念、完善人防体系，加强国防应急动员和国防后备力量建设。加强信访工作，畅通信访渠道，解决民众困难。可持续发展：稳定低生育水平、优化人口结构、提高人口素质、关注人口安全；落实资源保护政策、合理开发自然资源；不断改善环境质量，促进人与自然、社会和谐发展。

（六）坚持公平公正、服务至上，提高行政效能。以“保持共产党员先进性教育活动”为契机，围绕“科学行政、民主行政、依法行政”要求，发挥党员干部的模范带头作用，做到“遵章办事、和衷共事、自律行事、共谋大事”，打造“求真务实、廉洁高效”政府，重点是转变职能、依法行政、提高效率、勤政廉政。转变职能：按照“宏观调控、市场监管、社会管理、公共服务”职责，进一步加快政企分开、政事分开，进一步理顺部门职责关系，确保政府工作“不缺位”、“不越位”、“不错位”。依法行政：加强行政立法，严格依法行政，强化行政监督。自觉接受人大法律监督、工作监督、政协民主监督和社会舆论监督，认真办理人大代表议案、建议和政协提案，重视发挥各民主党派、工商联、各群众团体作用。提高效率：精减审批事项，减化审批程序，缩短审批时限，加快公文流转，提高服务水平。勤政廉政：强化廉洁自律，严格工作纪律，加强制度建设，加大违法违纪惩处力度。

各位代表：现在的南昌实力在增强、名声在扩大、地位在提升，回首过去——我们问心无愧；展望未来——我们信心百倍！在中部地区竞相崛起的浪潮中，省委、省政府对我们赋予了重托，450万南昌人民对我们寄予了厚望。我们一定要更加紧密团结在以胡锦涛同志为总书记的党中央周围，乘十六届四中全会东风，在市委的正确领导下，为做大做强南昌、实现快速崛起而奋起直追、策马扬鞭、一路高歌。
